# Study on the Durability of Bamboo Fiber Asphalt Mixture

**DOI:** 10.3390/ma14071667

**Published:** 2021-03-28

**Authors:** Chaoming Xia, Chaofan Wu, Kefei Liu, Kang Jiang

**Affiliations:** 1 Department of Road and Bridge Engineering, School of Civil Engineering, Central South University of Forestry & Technology, Changsha 410004, China; 20191200374@csuft.edu.cn; 2 Hunan Provincial Engineering Research Center for Construction Solid Wastes Recycling, Changsha 410205, China; cfwu0188@126.com; 3 Hunan Communications Research Institute Co. LTD, Changsha 410015, China

**Keywords:** durability, bamboo fiber asphalt mixture, grey correlation analysis, ageing durability, freeze-thaw cycle durability

## Abstract

To evaluate the durability of bamboo fiber asphalt mixture using four gradation schemes, the durability of the bamboo fiber asphalt mixture is studied considering three aspects: ageing durability, freeze-thaw cycle durability and fatigue durability through the Marshall test, indoor ageing test, uniaxial compression test, low-temperature bending test, immersion Marshall test, freeze-thaw splitting test and four-point bending fatigue test. Nonfiber asphalt mixture and lignin fiber asphalt mixture were used as control groups. The results show that the addition of plant fiber can effectively improve the durability of asphalt mixture. Bamboo fiber modified asphalt mastic has good ductility and adhesion due to its rough surface and good oil absorption performance. Bamboo fiber asphalt mixture has better and more stable low-temperature ageing durability and moisture ageing durability than lignin fiber asphalt mixture, but its mechanical property is weaker than the latter. The improvement effect of the two fibers on the freeze-thaw cycle durability of asphalt mixture is basically the same. Bamboo fiber can improve the flexibility of the mixture and delay the development of cracks so that the mixture has good fatigue durability. The smaller the void ratio, the thicker the asphalt film, and the denser the structure of the mixture, the better the durability. The durability of the stone mastic asphalt (SMA) gradation mixture is better than that of asphalt concrete (AC) gradation. The material composition and aggregate gradation of plant fiber asphalt mixture have a great influence on its durability. In the future, it is necessary to establish a multiparameter comprehensive evaluation index system among fiber type and properties, mixture gradation and durability so as to realize the directional regulation of the durability of different fiber asphalt mixtures. Bamboo fiber is a reliable substitute for lignin fiber, and further research on improving its surface properties and dispersion uniformity can be carried out in the future.

## 1. Introduction

The durability of asphalt mixture includes ageing durability, freeze-thaw cycle durability and fatigue durability. Asphalt ageing is an inevitable problem during the construction and the use of asphalt pavement, which has a significant impact on the pavement service life. Ageing can attenuate the performance of asphalt pavement, reduce its flexibility, resulting in pavement cracking, and ultimately aggravating the damage of pavement [1]. Under the joint action of low temperature and moisture, asphalt pavement can undergo serious freeze-thaw damage, resulting in the cracking, loosening, stripping, particle dropping, pumping and other damages, which will seriously affect the durability of asphalt pavement [2]. In addition, asphalt pavement is prone to fatigue failure under the repeated action of wheel load, manifesting as cracks along the longitudinal direction of the pavement, which then develops into net cracks and even potholes. This will not only affect driving comfort and increase operation and maintenance costs, but also reduce the service life of the pavement.

In an in-depth study on the durability of asphalt pavement, researchers found that adding fiber stabilizers in asphalt mixture can improve the crack resistance, ageing resistance, moisture damage resistance and durability of pavement and prolong the service life of roads. It is an effective measure to solve the performance defects of asphalt pavement [3,4]. At present, lignin fiber, mineral fiber and polymer fiber are widely used in asphalt mixture, among which lignin fiber is the most popular because of its good chemical stability, strong oil absorption capacity, and low price [5,6]. However, most lignin fibers are taken from logs, and their large-scale use does not conform to the development concept of resource conservation, so they are not suitable for large-scale application in the rapid growth of road infrastructure. Therefore, it is urgent to find green and renewable plant fibers to replace them.

China is a country lacking forestry resources. The per capita forest area and per capita forest stock are far lower than the world average [7]. On the other hand, China has the most abundant bamboo resources in the world [8]. Bamboo fiber has good physical and mechanical properties, which are stable and show little variation [9]. Furthermore, bamboo fiber is also cheap, recyclable, degradable and renewable, and the best material to replace lignin fiber for road use. Therefore, the use of fast-growing bamboo to replace conifer wood for road-used lignin fibers can alleviate the discrepancy between supply and demand of wood and realize the sustainable development of forest resources, and it is also an inevitable requirement for the development of the circular economy and the construction of a resource-saving and environmentally friendly society.

Great progress has been made in the durability research of road fiber and building materials. Li et al. [10] and Panda et al. [11] found that the addition of corn straw fiber and coconut fiber can effectively improve the road performance of asphalt mixture. Chen et al. [12] proved that the addition of short cut basalt fiber can effectively improve the fatigue durability of asphalt mixture. Zhang et al. [13] studied the durability of composite fiber modified asphalt mixture under salt freeze-thaw cycle conditions, and the results showed that composite fiber composed of polyester fiber, basalt fiber and sepiolite fiber can significantly improve the durability of asphalt pavement. Saeed et al. [14] evaluated the durability of aramid pulp fiber asphalt mixture, and the results showed that the addition of aramid pulp fiber effectively improved the fatigue life, thermal performance and durability of asphalt mixture under repeated freeze-thaw cycles. Khotbehsara et al. [15] found that a filler composed of fly ash and flame retardant can improve the durability of an epoxy resin system under hot and humid conditions. The test results of Li et al. [16] showed that the mechanical properties, crack resistance and durability of well cement can be significantly improved by using nanocarbon material as filler. Sheng et al. [17] found that the use of bamboo fiber can improve the rutting resistance and low-temperature crack resistance of asphalt mixture, and the improvement effect of bamboo fiber is better than that of polymer fiber and lignin fiber. Li [18] found that the addition of bamboo fiber improved the high temperature stability and low-temperature crack resistance of asphalt concrete. It can be seen that the current studies on bamboo fiber asphalt mixture are few and mostly focus on conventional road performance and the research on durability is still blank. Additionally, the existing literature on the durability of fiber asphalt mixture is not systematic and comprehensive enough, and the quantitative analysis of the factors affecting the durability of fiber asphalt mixture is also lacking.

To evaluate the durability of bamboo fiber asphalt mixture, flocculent bamboo fiber extracted from bamboo stem was mixed into asphalt mixture and compared with nonfiber asphalt mixture and lignin fiber asphalt mixture in this paper. A series of laboratory tests were conducted to study the durability of bamboo fiber asphalt mixture from three aspects of ageing durability, freeze-thaw cycle durability and fatigue durability, and the grey correlation theory was used to evaluate the factors affecting the durability of asphalt mixture. This in-depth study on the effects and mechanisms by which bamboo fiber improves the durability of asphalt mixture can not only provide a design basis for the engineering application of plant fiber asphalt mixture, but also effectively improve the road performance, service life and service level of asphalt pavement.

## 2. Materials and Methods

### 2.1. Materials

#### 2.1.1. Asphalt

The asphalt used is styrene-butadiene-styrene (SBS) modified asphalt, produced by Changlian Petrochemical Co., Ltd. (Hunan, China). Its basic properties are shown in Table 1.

#### 2.1.2. Fibers

Two types of plant fibers, lignin fiber and bamboo fiber, were employed to prepare the fiber-reinforced asphalt mixtures in this study. The contents of both types of fiber in asphalt mastic were set at 1.0 wt%, and their contents in asphalt mixture were determined by the results of the Marshall stability test (detailed later).

**Figure 1:**
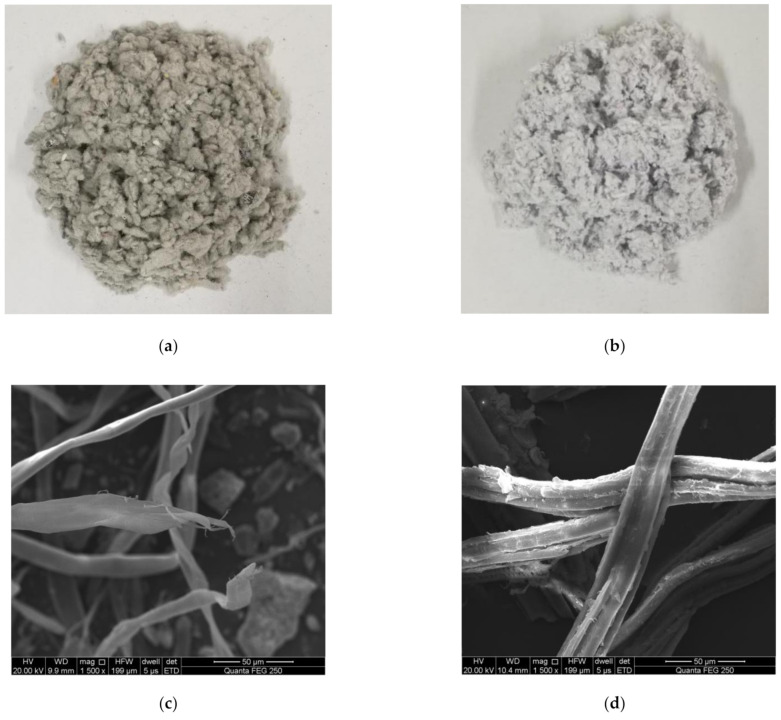
Digital photos of (**a**) lignin fiber and (**b**) bamboo fiber showing their difference in appearance vs. scanning electron microscope (SEM) micrographs of (**c**) lignin fiber and (**d**) bamboo fiber revealing their microstructures at the magnification of 1500×.

The lignin fiber was a flocculent fiber provided by Galway Technology Co., Ltd. (Sichuan, China), whereas the bamboo fiber was a flocculent fiber (Homemade, Changsha, Hunan, China) fabricated in laboratory. The subtle differences between the two fibers can be observed from their digital photos (Figure 1a,b) as well as from their scanning electron microscope (SEM, Quanta, Houston, TX, USA) micrographs (Figure 1c,d). The SEM micrographs reveal that lignin fibers (with typical lengths of 4 to 10 mm) have smooth surfaces and relatively uniform diameters across single fibers (typically 10 to 20 µm) and they interweave with prominent branches at the ends, which cause lap bonding and enhance the adhesion between asphalt and aggregate. In contrast, bamboo fibers (with typical lengths of 4 to 8 mm) have diameters that are more diverse across single fibers (typically 15 to 20 µm) and feature the presence of gullies on the fiber surface.

Table 2 presents the basic technical properties of each fiber, suggesting the similarities and differences between the two fibers. It can be seen from Table 2 that each index of the two fibers meets the requirement of *Plant fibers used in asphalt pavements* (JT/T 533-2004 [19]). Furthermore, bamboo fiber has a higher oil absorption and lower water absorption than the lignin fiber, implying that the bamboo fiber has better potential to absorb asphalt binder and resist the ingress of moisture.

#### 2.1.3. Aggregates

Basalt (Qingshan, Hengyang, Hunan, China) and limestone chips (Xinri, Hengyang, Hunan, China) were used as the coarse aggregate and fine aggregate, respectively, and Table 3 presents the properties of each aggregate. The mineral filler is limestone powder. All the properties of aggregates and mineral filler meet the technical requirements of specification JTG F40-2004 [20], as detailed in Table 3.

### 2.2. Sample Preparation

The gradation schemes used in this article include AC-13, AC-16, SMA-13 and SMA-16. Based on the Marshall mix ratio test, the aggregate gradations are shown in Table 4, and the mix design data of each asphalt mixture are shown in Table 5.

### 2.3. Experiments

#### 2.3.1. Physical Properties Test

The penetration, softening point, ductility and viscosity of the fiber asphalt mastic were tested according to JTG E20-2011 [21].

#### 2.3.2. Marshall Test

The contents of plant fiber were 0.2 wt%, 0.3 wt%, 0.4 wt%, 0.5 wt% and 0.6 wt% of the total mass of asphalt mixture, respectively. According to the test specification JTG E20-2011, Marshall specimens of four gradations of asphalt mixture were prepared (Marshall compactor, Tuoxing, Nanjing, Jiangsu, China). The optimum fiber content was determined according to the requirements of technical specifications. To test each index and calculate the optimum asphalt content of each fiber asphalt mixture, the specific results are shown in Table 6.

#### 2.3.3. Ageing Test

The thermal oxidative ageing performance of fiber asphalt mixture was tested by controlling ageing time and ageing temperature (Oven, Quannai, Changzhou, Jiangsu, China). Under the condition of forced ventilation, different asphalt mixtures were heated and aged by oven. When the test temperature was 80 °C, the ageing times were 0, 30, 60, 90 and 120 h. When the ageing time was 120 h, the test temperatures were 80, 90, 100, 110 and 120 °C.

#### 2.3.4. Mechanical Properties Test

The compressive strength and compressive resilient modulus of asphalt mixture before and after ageing at 20 °C were tested to evaluate its mechanical properties. A cylindrical specimen of φ100 mm × 100 mm was formed by static pressure method (Static pressure forming instrument, Taiding, Cangzhou, Hebei, China). MTS-810 universal material testing machine (MTS, Eden Prairie, MN, USA) was used for the test and the loading rate was 2 mm/min.

#### 2.3.5. Low-Temperature Bending Test

The trabecular bending test (Bending tester, Changji, Shanghai, China) was used to evaluate the low-temperature stability of asphalt mixture before and after ageing; the bending stiffness modulus and the maximum bending strain of fiber asphalt mixture were measured at −10 °C. The sample was cut into prismatic trabeculars of 250 mm × 30 mm × 35 mm and the loading rate was 50 mm/min.

#### 2.3.6. Moisture Stability Test

The moisture stability of asphalt mixture before and after ageing was evaluated by immersion Marshall test (Marshall compactor, Tuoxing, Nanjing, Jiangsu, China) and freeze-thaw splitting test (Splitting tester, Zhulong, Cangzhou, Hebei, China); the immersion Marshall residual stability and freeze-thaw splitting strength of fiber asphalt mixture were tested, respectively. Freeze-thaw cycle durability was evaluated by a freeze-thaw splitting test under different freeze-thaw cycles. The conditions of a single freeze-thaw cycle were as follows: the sample was frozen at −18 °C for 16 h and then placed in a thermostatic water tank (Jiangnan, Ningbo, Zhejiang, China) at 60 °C for 24 h. Each sample was freeze-thawed 1, 2, 3, 4 and 5 times, respectively. All freeze-thaw cycle samples were put in a constant temperature water tank at 25 °C for 2 h, and then the splitting tensile strength was measured by an SYD-0731 uniaxial compression testing machine (Meiyu, Shanghai, China), and the splitting tensile strength ratio was calculated.

#### 2.3.7. Four-Point Bending Fatigue Test

The fatigue life of fiber asphalt mixture was measured by a four-point trabecular bending fatigue test (MTS, Eden Prairie, MN, USA). The sample size was 380 mm × 63 mm × 50 mm, test temperature was 15 °C, loading frequency was 10 Hz, and the loading waveform was continuous sine wave. The stress control mode was adopted, and the stress levels were 0.3, 0.4 and 0.5 of the flexural tensile strength of each mixture, respectively. Three parallel specimens were taken for testing at each level, and the test results were averaged.

**Figure 2:**
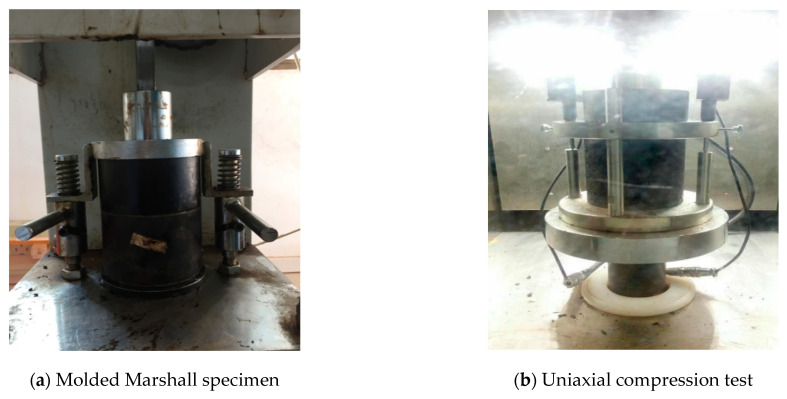

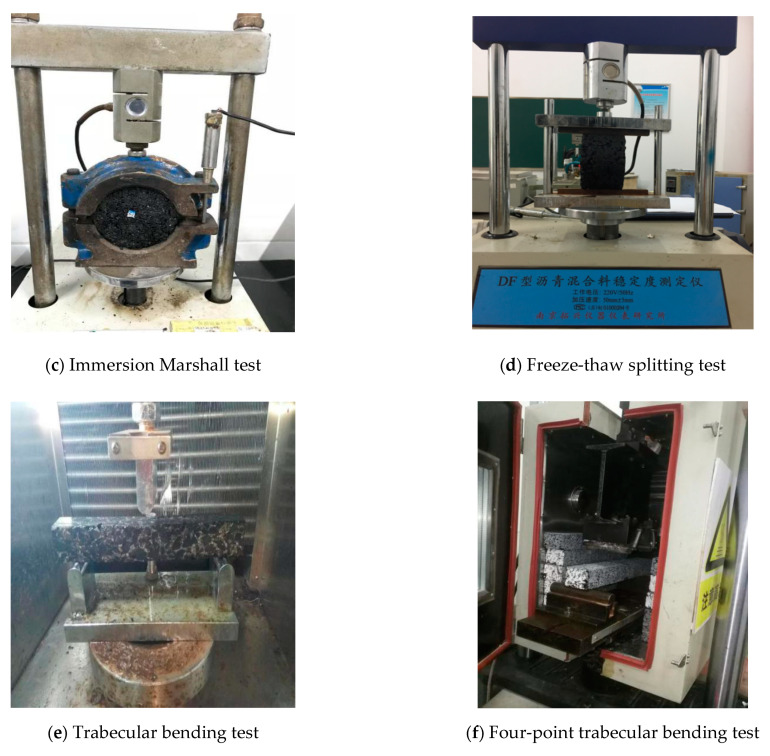
Setup for each test.

The setup for each test is shown in Figure 2.

## 3. Results and Discussion

### 3.1. Physical Properties of Fiber Asphalt Mastic

The test results demonstrating physical properties of each fiber asphalt mastic are shown in Table 7.

It can be seen from Table 7 that the penetration of lignin fiber and bamboo fiber asphalt mastic decreased by 8.8% and 3.2%, respectively, compared to that of nonfiber asphalt mastic, indicating that the addition of fiber improves the consistency of the asphalt and thus improves its deformation resistance. The ductility of nonfiber asphalt mastic is 11.1% and 3.3% higher than that of lignin fiber and bamboo fiber asphalt mastic, respectively, which proves that the low-temperature crack resistance of asphalt mastic cannot be improved by adding fiber, but will slightly decrease. The viscosities of lignin fiber and bamboo fiber asphalt mastic are 12.3% and 14.7% higher than that of nonfiber asphalt mastic, showing that plant fiber can increase the internal friction resistance of asphalt flow and its viscosity. The corresponding elastic recovery rates of the two kinds of fiber asphalt mastic are 8.6% and 12.3% higher than that of nonfiber asphalt mastic, respectively, demonstrating that the addition of plant fiber can improve the toughness of asphalt mastic, improve its self-healing ability after shape change, and then improve the fatigue performance of asphalt mastic [22]. Bamboo fiber has a better improvement effect.

### 3.2. Ageing Durability

#### 3.2.1. Different Ageing Times

##### Mechanical Properties

**Figure 3:**
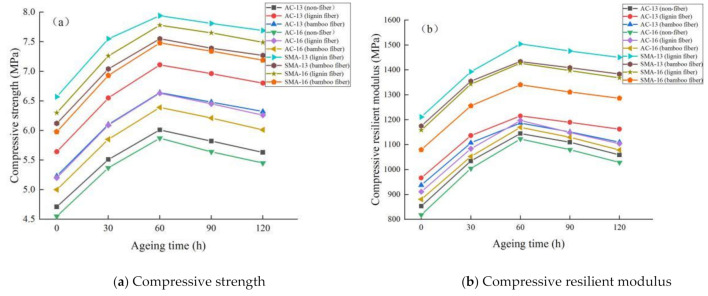
Uniaxial compression test results of fiber asphalt mixture under different ageing times.

The uniaxial compression test results of fiber asphalt mixture under different ageing times are shown in Figure 3.

**Figure 4:**
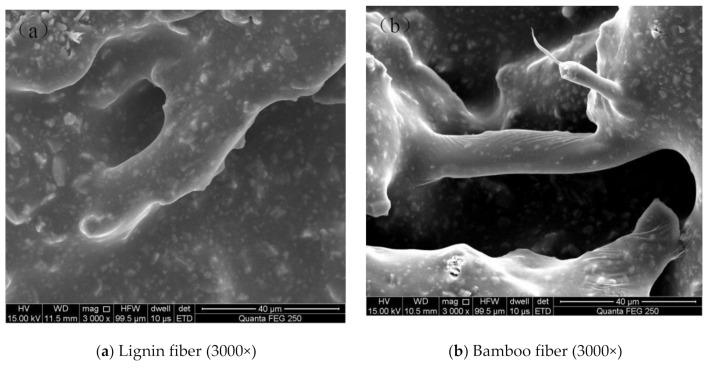
“Reinforcement” and “bridging” effects of two kinds of fibers in asphalt mixture.

It can be seen from Figure 3, that:

(1) with the extension of ageing time, the compressive strength and compressive resilient modulus of fiber asphalt mixture first increase and then decrease, indicating that short-term ageing can improve the mechanical properties of asphalt mixture, but with the increase in ageing, the mechanical properties decrease slightly. The possible reason for this is that the light oil in asphalt volatilizes at the early stage of ageing, so the content of oxygen-containing polar functional groups in asphalt increases, the asphalt becomes hard and brittle, and its antideformation ability improves [23]. However, with the extension of ageing time, the structure of asphalt mixture is destroyed and its mechanical properties are reduced.

(2) compared with the nonfiber asphalt mixture, the addition of plant fiber can effectively improve the compressive resilient modulus and compressive strength of asphalt mixture, and the mechanical properties are better. On the one hand, the addition of fiber can adsorb polar molecules in asphalt and inhibit its volatilization and strengthen the bond between aggregate and asphalt, reduce the opportunity of interaction between asphalt and oxygen, slow down the conversion of light components to asphaltenes, and delay the ageing process [24]. On the other hand, the addition of an appropriate amount of fiber in the asphalt mixture can aid in bridging and reinforcement (see Figure 4), so that the external load is evenly dispersed in the aggregate and asphalt mastic, and the structure stress is more uniform, avoiding the phenomenon of stress concentration, thus improving the compressive strength of asphalt mixture. In addition, after the asphalt mixture is loaded, the fibers dispersed in it can improve the resilience of the asphalt mixture and reduce the resilient deformation [25].

(3) under the same gradation, the compressive resilient modulus and compressive strength of lignin fiber asphalt mixture are greater than that of bamboo fiber, demonstrating that the lignin fiber asphalt mixture has better mechanical properties before and after ageing. This may be due to the fact that bamboo fiber has relatively few branches at the end, and its ability to disperse the load and dissipate the strain energy is slightly weak when subjected to loads after ageing.

(4) using the same fiber, the order of compressive resilient modulus and compressive strength of asphalt mixtures with different gradations is SMA-13 > SMA-16 > AC-13 > AC-16, in descending order from large to small. The SMA gradation mixture has a thicker asphalt film, a small void ratio, and less contact between aggregates and air, so the mixture has good ageing resistance. Furthermore, more coarse aggregates in SMA gradation allow intercalation to be maximized, and the fine aggregates have higher cohesion, so the mechanical property of the whole structure is better. Overall, the more fine aggregate content in the mixture, the denser the structure and the better the mechanical properties.

##### Low-Temperature Performance

**Figure 5:**
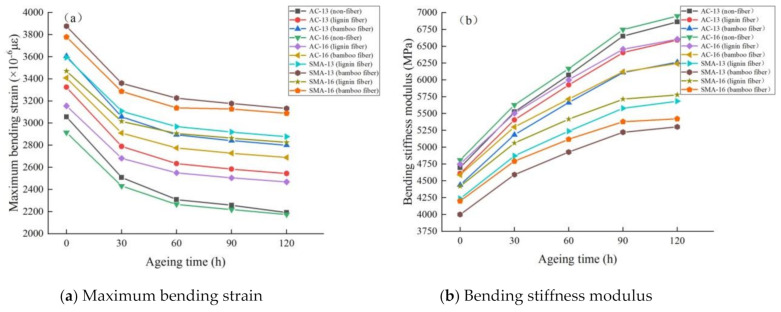
Low-temperature performance test results of fiber asphalt mixture under different ageing times.

The test results demonstrating low-temperature performance of fiber asphalt mixture under different ageing times are shown in Figure 5.

It can be seen from Figure 5 that:

(1) with the extension of ageing time, the maximum bending strain of fiber asphalt mixture decreases, while the bending stiffness modulus increases. In the ageing process, asphalt undergoes oxidation, volatilization and polymerization, resulting in the decrease in light components and the increase in heavy components [26]; the asphalt binder becomes brittle and hard, and its antideformation ability decreases. As a result, the bending stiffness modulus of asphalt mixture increases, the bending strain decreases, and as it is easy to fracture at low temperatures, the low-temperature crack resistance becomes worse.

(2) compared with the nonfiber asphalt mixture, the maximum bending strain of plant fiber asphalt mixture is larger, the bending stiffness modulus is smaller, and the change rate is smaller, indicating that the addition of fiber can improve the low-temperature bending ageing durability of asphalt mixture. In fact, the short-cut flocculent fibers have good dispersibility in the asphalt mixture and are easy to overlap to form fiber asphalt mastic structure. Bamboo fiber itself has good tensile strength, pull-out strength and ageing resistance, and is not easy to be separated from asphalt molecules, which can prevent the generation and expansion of cracks under low-temperature loads [27]. In addition, a large number of fibers and asphalts coated with each other increases the thickness of asphalt film, which not only inhibits the ageing of asphalt, but also reduces the content of “free asphalt” and improves the integrity of asphalt mixture.

(3) under the same gradation, the maximum bending strain of bamboo fiber asphalt mixture is higher than that of lignin fiber, and the bending stiffness modulus is smaller than that of lignin fiber, showing that bamboo fiber asphalt mixture has stronger toughness and low-temperature cracking resistance. The main reason for this is that the surface of bamboo fiber is rough (as shown in Figure 1) and the length is different, which has a strong bonding force with the surface of asphalt, and enhances the toughness of the asphalt mastic, thereby increasing the antideformation ability of asphalt mixture. The surface of lignin fiber is relatively smooth, and its binding with asphalt is relatively weak. In addition, the change rate of low-temperature performance parameters of bamboo fiber asphalt mixture in the ageing process is lower than that of lignin fiber, indicating that its low-temperature ageing durability is better and more stable. As can be seen from Table 5, bamboo fiber asphalt mixture has a higher asphalt consumption, and its high asphalt content can form a thicker asphalt film than lignin fiber asphalt mixture, thus enhancing the ductility of the mixture and improving the low-temperature stability of the mixture in the ageing process.

(4) using the same fiber, the order of low-temperature crack resistance of asphalt mixtures with different gradations is SMA-13 > SMA-16 > AC-13 > AC-16, in descending order from large to small. The skeleton voids of the SMA mixture are filled with a large amount of asphalt and completely coated on the surface of aggregate. Additionally, the SMA mixture with more fine aggregates has a larger specific surface area, and the adsorption effect of asphalt is more obvious, which shows that the asphalt mastic has a stronger bonding effect on the aggregate. Therefore, the SMA mixture has stronger antiageing properties and low-temperature deformation resistance. However, during the ageing process, the degradation, rate of low-temperature crack resistance of four kinds of mixtures from large to small is AC-13 > AC-16 > SMA-13 > SMA-16. The results show that the more the fine aggregate content and the larger the specific surface area in the mixture, the more easily its low-temperature performance is affected by ageing and the faster the low-temperature performance declines.

##### Moisture Stability

**Figure 6:**
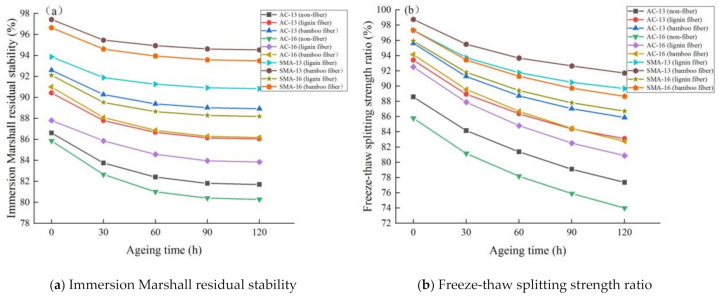
Test results of moisture stability of fiber asphalt mixture under different ageing times.

The results of moisture stability test of fiber asphalt mixture under different ageing times are shown in Figure 6.

It can be seen from Figure 6 that:

(1) with the extension of ageing time, the immersion Marshall residual stability of fiber asphalt mixture decreases continuously and the moisture stability becomes worse. This is because the ageing of asphalt reduces its adhesion to the aggregate, and moisture gradually intrudes between them, weakening the bond between asphalt and aggregate, resulting in the increase in void ratio of the mixture and loss of cohesive force. The spalling of the asphalt film causes the aggregate to be damaged due to exposure, which reduces the integrity and strength of structure and deteriorates the mechanical properties. Moreover, oxygen-containing functional groups such as ketones and acids with strong hydrophilicity will be generated during the ageing process of asphalt, which greatly increases the solubility of asphalt molecules in water and aggravates the moisture damage of asphalt mixture [28].

(2) compared with the nonfiber asphalt mixture, the *MS*_0_ and *TSR* values of the fiber asphalt mixture are higher, and the change rate are less than that of the nonfiber asphalt mixture, indicating that the addition of fibers can improve and stabilize the moisture ageing durability of asphalt mixture. The distribution of plant fibers in asphalt can form a three-dimensional network of asphalt mastic, which can bind the mixture into a solid whole and effectively reduce the impact of ageing on the moisture stability of asphalt mixture. Furthermore, the oil absorption of fiber can delay the penetration of water, reduce the contact between asphalt film and water, slow down the process of moisture damage after asphalt mixture ageing, and improve the ability of asphalt mixture to resist freeze-thaw and moisture damage.

(3) during the ageing process, the *MS*_0_ and *TSR* values of the bamboo fiber asphalt mixture were higher than that of lignin fiber, showing that the bamboo fiber asphalt mixture has better moisture stability. The change rate of the moisture stability parameter of bamboo fiber asphalt mixture is lower than that of lignin fiber, which indicates that bamboo fiber asphalt mixture has better moisture stability and ageing durability. For one thing, bamboo fiber asphalt mixture has a higher asphalt content, so the asphalt film coated with aggregate is also thickened, thus effectively reducing the oxidation of polar substances in the ageing process [29]. Additionally, the water absorption of bamboo fiber is slightly lower than that of lignin fiber. The greater the water absorption of the fiber, the more prone to wedge erosion and wet swelling with the asphalt surface with moisture invasion, which is similar to the spalling phenomenon of the asphalt-aggregate surface, so that the ability of asphalt mixture resistance to moisture damage is weakened.

(4) using the same fiber, the *MS*_0_ and *TSR* values of asphalt mixture with different gradations are sorted from large to small as SMA-13 > SMA-16 > AC-13 > AC-16, and the order of change rate is just the opposite. This demonstrates that the gradation SMA-13 is the best, followed by SMA-16 and AC-13, and AC-16 is the worst in terms of moisture stability and durability. SMA mixture is fully filled with asphalt mastic and has less contact with air, and has a thicker asphalt film that can reduce the damage of moisture to the structure, so it has good ageing resistance. Additionally, the larger the aggregate particle size and the larger the waterproof void, the worse the adhesion, resulting in the poor resistance to moisture damage.

#### 3.2.2. Different Ageing Temperatures

##### Mechanical Properties

**Figure 7:**
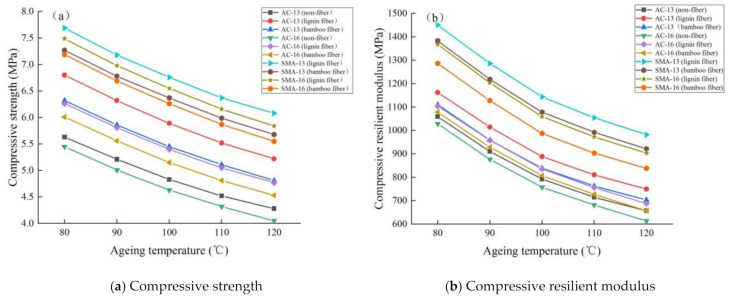
Test results of mechanical properties of fiber asphalt mixture under different ageing temperatures.

The test results demonstrating mechanical properties of fiber asphalt mixture at different ageing temperatures are shown in Figure 7.

It can be seen from Figure 7 that:

(1) with the increase in ageing temperature, the compressive resilient modulus and compressive strength of fiber asphalt mixture show a decreasing trend, which indicates that the increase in ageing temperature can reduce the mechanical properties of asphalt mixture. This is because the higher the ageing temperature, the more easily polar molecules in asphalt react with oxygen. Meanwhile, the viscosity of asphalt decreases, the resistance of intermolecular binding weakens, and the adhesion between asphalt and aggregates weakens. Therefore, the mechanical properties of asphalt mixture decrease at higher ageing temperatures [30].

(2) compared with nonfiber asphalt mixture, plant fiber asphalt mixture has higher compressive strength and compressive resilient modulus, smaller change rate and better mechanical properties. The increase in ageing temperature can significantly decrease the adhesion between asphalt and aggregate [31], and the fibers present three-dimensional distribution in the asphalt mixture, which forms vertical and horizontal interwoven spatial networks by overlapping each other, which is conducive to improving the adhesion and adsorption between asphalt mastic and aggregate.

(3) under the same gradation, the compressive strength and compressive resilient modulus of lignin fiber asphalt mixture are higher than that of bamboo fiber, but the change rate is lower than the latter. This result is consistent with Section 3.2.1.

(4) using the same fiber, the order of mechanical properties of asphalt mixture with different gradations from large to small is SMA-13 > SMA-16 > AC-13 > AC-16, and the order of change rate is completely the opposite, which proves that SMA-13 has the best durability of asphalt mixture with different ageing temperatures, followed by SMA-16 and AC-13, and AC-16 is the worst. This result is consistent with Section 3.2.1.

##### Low-Temperature Performance

**Figure 8:**
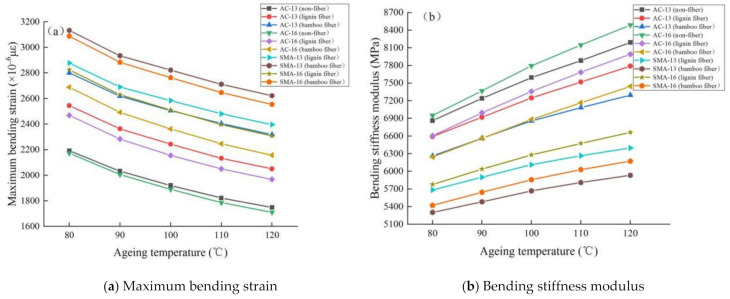
Low-temperature performance test results of fiber asphalt mixture under different ageing temperatures.

The test results demonstrating low-temperature performance of fiber asphalt mixture at different ageing temperatures are shown in Figure 8.

It can be seen from Figure 8 that:

(1) the bending stiffness modulus of asphalt mixture increases with the increase in ageing temperature, while the reverse is true for the maximum bending strain. This shows that the low-temperature stability of asphalt mixture deteriorates with the increase in ageing temperature. The main reason for this is that a high temperature accelerates the volatilization of the light components of asphalt and the oxidation process of asphaltenes, which causes the asphalt to become brittle and hard, thereby reducing the flexibility of asphalt and weakening the ability to resist low-temperature load.

(2) the low-temperature bending performance and parameter change rate of plant fiber asphalt mixture are lower than those of nonfiber asphalt mixture, and the low-temperature stability is better. This result is consistent with Section 3.2.1.

(3) under different ageing temperatures, the maximum bending strain of bamboo fiber asphalt mixture is higher than that of lignin fiber, while the bending stiffness modulus of bamboo fiber asphalt mixture and the change rate of two parameters are lower than those of lignin fiber, illustrating that the low-temperature ageing durability of bamboo fiber asphalt mixture is better and more stable than that of lignin fiber at different ageing temperatures.

(4) using the same fiber, the order of the maximum bending strain of four gradation asphalt mixtures from large to small is SMA-13 > SMA-16 > AC-13 > AC-16, while the order of bending stiffness modulus is the opposite. During the process of increasing ageing temperature, the change rate of low-temperature performance parameters of each asphalt mixture is ranked as AC-16 > AC-13 > SMA-16 > SMA-13. This shows that the SMA-13 gradation has the best low-temperature bending durability at different ageing temperatures, followed by SMA-16 and AC-13, and AC-16 is the worst. In general, the denser the structure of the mixture, the less it is affected by the ageing temperature, and the stronger the low-temperature stability after ageing.

##### Moisture Stability

**Figure 9:**
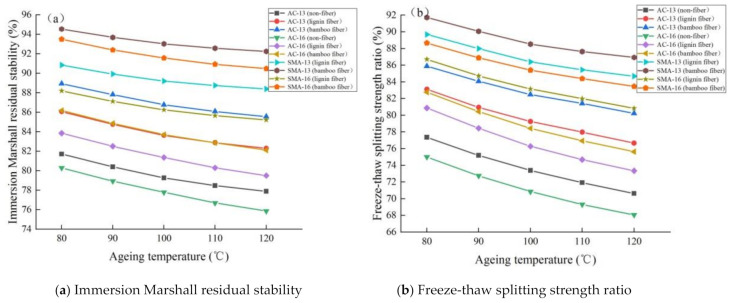
Results of moisture stability test of fiber asphalt mixture under different ageing temperatures.

The results of moisture stability test of fiber asphalt mixture at different ageing temperatures are shown in Figure 9.

It can be seen from Figure 9 that:

(1) with the increase in ageing temperature, the *MS*_0_ and *TSR* values of the fiber asphalt mixture showed a decreasing trend, and the moisture stability becomes worse. This is because the increase in ageing temperature leads to the deepening of ageing degree, and the hydrophilic substances in asphalt components and stiffness modulus of asphalt mixture increase, resulting in the swelling damage of asphalt mixture during freeze-thaw cycle and the significant attenuation of the *TSR* value. Additionally, the increase in ageing temperature leads to the decrease in asphalt bonding force, so the moisture can more easily to enter into the mixture, and the moisture stability of the mixture decreases.

(2) the moisture stability of plant fiber asphalt mixture is better than that of nonfiber asphalt mixture at different ageing temperatures, and the change rates of parameters are also smaller. This result is consistent with Section 3.2.1.

(3) with the increase in ageing temperature, the *MS*_0_ and *TSR* values of bamboo fiber asphalt mixture are higher than that of lignin fiber, but the change rates of parameters are lower than the latter. This shows that bamboo fiber asphalt mixture has better moisture stability durability under different ageing temperatures.

(4) under the same fiber, the moisture stability parameters of asphalt mixtures with different gradations are sorted from large to small as SMA-13 > SMA-16 > AC-13 > AC-16, and the change rates are completely the opposite. This result is consistent with Section 3.2.1.

### 3.3. Freeze-Thaw Cycle Durability

**Figure 10:**
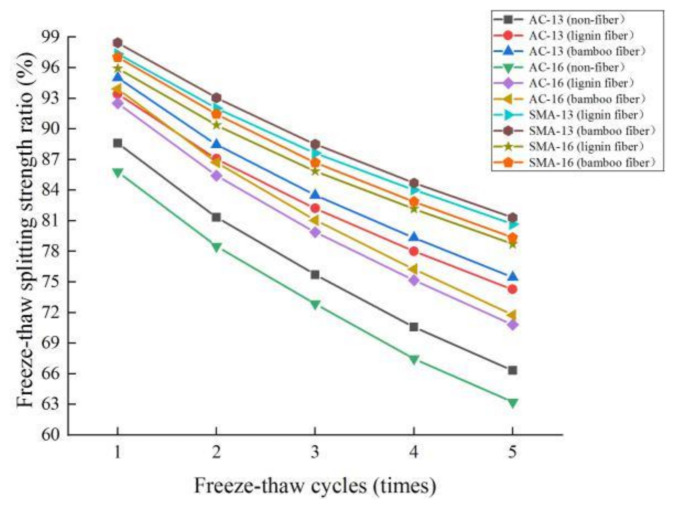
*TSR* test results of fiber asphalt mixture under different freeze-thaw times.

The test results demonstrating the freeze-thaw splitting tensile strength ratio of fiber asphalt mixture under different freeze-thaw cycles are shown in Figure 10.

It can be seen from Figure 10 that:

(1) with the increase in freeze-thaw cycles, the splitting tensile strength of fiber asphalt mixture decrease significantly. The process of a freeze-thaw cycle includes frost heaving and water erosion. The frost heaving is obvious in the early stage of the freeze-thaw cycle, while the water erosion runs through the whole freeze-thaw cycle [32]. In the process of frost heaving, the pore water expands and freezes at a low temperature, resulting in a frost heaving effect on the pore wall. Asphalt shows elasticity brittleness, and cracks are initiated under frost heaving force, which increases the porosity and expands the pores, so the asphalt mixture exhibits cohesive failure. In the process of water erosion, water adsorbs on the surface of asphalt to constantly replace asphalt, which makes it easier to peel off and cause adhesion failure. Under the action of repeated freeze-thaw cycles, the road performance of asphalt mixture decreases rapidly.

(2) compared with nonfiber asphalt mixture, the *TSR* value of fiber asphalt mixture is larger and the reduction rate is slower, which indicates that the addition of fiber can effectively improve the freeze-thaw cycle durability of asphalt mixture. In the plant fiber mixture, a large number of randomly dispersed fiber networks connect the structure into a stronger whole, the binding force between asphalt and aggregates increases, and the erosion effect of moisture decreases. In addition, fiber can absorb asphalt, making the structural asphalt film thicker, and effectively slowing down the stripping of asphalt and aggregate in asphalt mixture under the action of water.

(3) with the same gradation, the *TSR* value of bamboo fiber asphalt mixture is slightly higher than that of lignin fiber, but the change rate is basically the same, demonstrating that bamboo fiber and lignin fiber have the same effect on improving the freeze-thaw cycle durability of asphalt mixture.

(4) using the same fiber, the order of *TSR* values of four gradation asphalt mixtures from large to small is SMA-13 > SMA-16 > AC-13 > AC-16, and the decline rate of the *TSR* value shows the opposite trend. It shows that in terms of freeze-thaw cycle durability, the SMA-13 gradation is the best, followed by SMA-16 and AC-13, and AC-16 is the worst. Good freeze-thaw cycle durability requires not only sufficient coarse aggregates to form a skeleton, but also dense filling and an appropriate void ratio [33]. SMA-type gradation has fewer intermediate aggregates, and the content of coarse aggregates (>4.75 mm) is more than 72.3%. It can form a strong skeleton and is filled with sufficient fine aggregates, so it has good frost resistance. The skeleton effect of AC-type gradation is weak, and the filling effect of fine aggregate on voids is not as dense as SMA-type gradation, so the frost resistance of AC mixture is weaker than that of SMA mixture.

### 3.4. Fatigue Durability

The fatigue equation of asphalt mixture under stress control mode is shown as follows [34]:

(1) $$N_{f}=K{\left(\frac{1}{\sigma_{0}}\right)}^{n}$$

where, *N_f_* is the fatigue life (times), *σ***_0_** is the initial bending stress (MPa), and *K* and *n* are the fatigue equation parameters determined by fatigue test.

The logarithm on both sides of Equation (1) was used to get:

(2) $$lgN_{f}=-nlg\sigma_{0}+lgK$$

where *K* is the height of the fatigue test curve; the higher the *K* value, the stronger the ability of asphalt mixture to resist repeated load. *n* is the slope of fatigue test curve; the greater the *n* value, the more easily affected the fatigue life of asphalt mixture is by stress.

**Figure 11:**
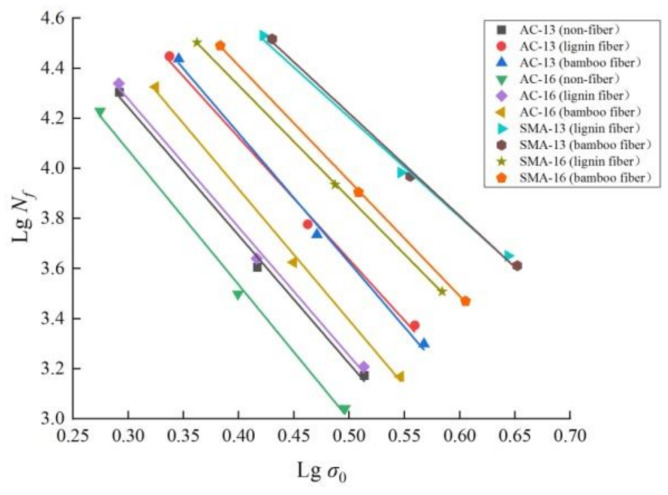
Relationship between fatigue life and stress level of asphalt mixture with different fibers.

The fatigue life analysis results of fiber asphalt mixture with the change of stress are shown in Figure 11, and the regression equation of the fatigue life of fiber asphalt mixture is shown in Table 8.

From the above analysis results, it can be seen that:

(1) the linear decreasing relationship between lg*N_f_* and lg*σ***_0_** indicates that the fatigue life of plant fiber asphalt mixture decreases with the increase in loading stress. Under the action of fatigue load, internal cracks initiate inside the mixture structure, stress concentration occurs at the initial defects and microcracks expand at a faster rate. The repeated action of the load further expands the cracks and finally leads to structural failure.

(2) the *K* value of plant fiber asphalt mixture at each stress level is greater than that of nonfiber asphalt mixture, and the *n* value is less than that of latter, indicating that the addition of fiber can improve the fatigue durability of asphalt mixture. The main reasons are as follows: firstly, the irregular distribution of fibers can restrain the crack and prevent further spreading of the crack. In addition, the slender filamentous plant fibers connect the whole structure, which can cause a certain tensile effect to maintain the integrity of the entire structure when the aggregates in the asphalt mixture separate [35]. Secondly, asphalt itself has certain self-healing ability of microcracks, and the tensile strength of fiber is higher than that of asphalt substrate. Under the action of external load, fiber can enhance the hysteretic recovery ability of asphalt mixture [36].

(3) under each stress level, the *K* value of bamboo fiber asphalt mixture is greater than that of lignin fiber, proving that bamboo fiber asphalt mixture has better resistance to repeated load. The improvement degree of fatigue life of asphalt mixture by fiber is related to the dispersion degree of fiber in the mixture, the aspect ratio, density, toughness of fiber and the ability to absorb asphalt [37]. It can be seen from Table 2 that bamboo fiber is denser than lignin fiber, and it has higher strength and toughness, so the reinforcement effect on the mixture is more obvious. Furthermore, bamboo fiber has better oil absorption, and the increase in asphalt content can improve the flexibility of mixture, which is conducive to the filling and healing of fine cracks, thereby delaying the structural damage and effectively improving the fatigue performance of asphalt mixture.

(4) under each stress level, the *n* value of bamboo fiber asphalt mixture is slightly larger than that of lignin fiber, showing that the fatigue sensitivity of bamboo fiber asphalt mixture is slightly worse, but the difference is not significant.

(5) under each stress level, the order of *K* value of four gradation asphalt mixtures from large to small is SMA-13 > SMA-16 > AC-13 > AC-16. The order of *n* values is the exact opposite. This shows that the gradation SMA-13 is the best in terms of fatigue durability of mixture, followed by SMA-16 and AC-13, and AC-16 is the worst. In SMA gradation, coarse aggregates are close to each other to form a skeleton, the friction between aggregates is larger, and the fine aggregates have greater compactness and cohesion, which improves the fatigue performance of the mixture. However, coarse aggregates in AC gradation are fewer and are not in contact, and the internal friction is weak, so it is prone to rutting, pushing and other deformations when the load is large, which affects its fatigue resistance [38]. The lower the asphalt content, the worse the cohesion between it and aggregates, so in terms of the fatigue performance, SMA-16 is worse than SMA-13, and AC-16 is worse than AC-13.

### 3.5. Grey Correlation Analysis

The factors affecting the durability of fiber asphalt mixture are evaluated by grey correlation theory. In terms of ageing durability, compressive strength, maximum bending strain and *TSR* values under different ageing conditions are taken as evaluation indexes, and ageing temperature, ageing time, properties of fiber asphalt mastic and aggregate gradation are taken as influencing factors. In terms of freeze-thaw cycle durability, *TSR* value under different freeze-thaw times is taken as the evaluation index, and the freeze-thaw cycle times, properties of fiber asphalt mastic and aggregate gradation are taken as influencing factors. In terms of fatigue durability, the fatigue life is taken as evaluation index, and the stress ratio, properties of fiber asphalt mastic and aggregate gradation are taken as influencing factors. For each durability evaluation, five groups of data were randomly selected from the laboratory test results for correlation analysis. The values of evaluation indexes and influencing factors for the durability of each mixture are shown in Table 9 and Table 10.

**Figure 12:**
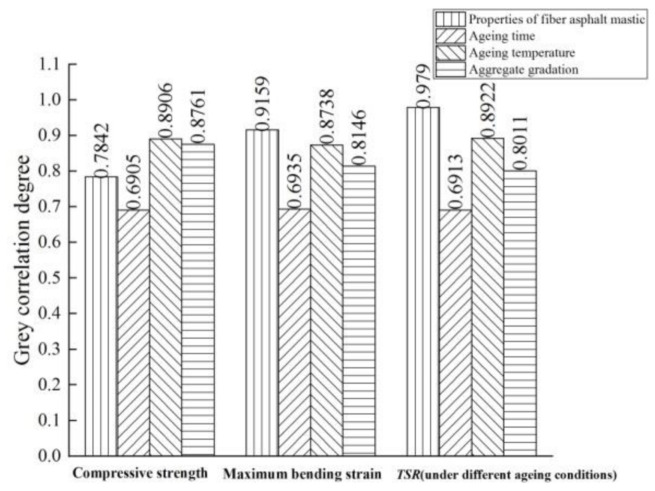
Grey correlation analysis results of influencing factors of ageing durability.

**Figure 13:**
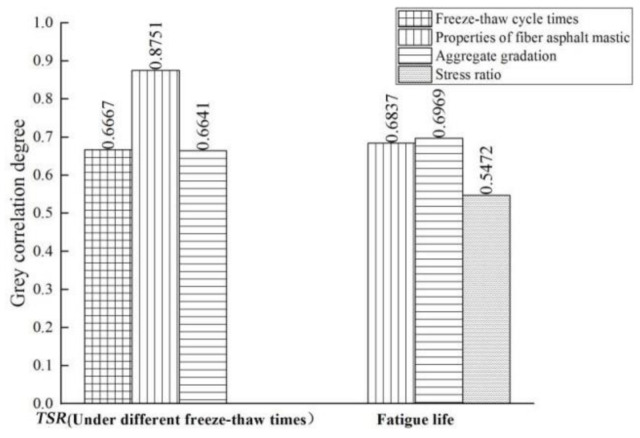
Grey correlation analysis results of influencing factors of freeze-thaw cycle and fatigue durability.

The grey correlation analysis results are shown in Figure 12 and Figure 13.

It can be seen from the figures that:

(1) in terms of mechanical properties, the influencing factors of ageing durability are ranked as follows: ageing temperature > aggregate gradation > properties of fiber asphalt mastic > ageing time. The correlation between ageing temperature and compressive strength is 0.8906, indicating that ageing temperature has the greatest influence on the mechanical ageing durability of asphalt mixture. The increase in ageing temperature intensifies the molecular activities and oxidation degree of asphalt in the ageing process, accelerates the ageing of asphalt mixture, and the mechanical properties of asphalt mixture gradually change from elastic to viscous. The bonding force between asphalt and aggregate weakens, the asphalt mixture gradually becomes soft, and the compressive strength and compressive resilient modulus decrease [39]. The correlation between aggregate gradation and compressive strength is 0.8761, which is the second major factor. The skeleton-type coarse aggregate content and asphalt content of asphalt mixture have important effects on its mechanical ageing durability.

In terms of low-temperature stability, the influencing factors of ageing durability are ranked as follows: properties of fiber asphalt mastic > ageing temperature > aggregate gradation > ageing time. The correlation between the properties of fiber asphalt mastic and the maximum bending strain is 0.9159, indicating that properties of fiber asphalt mastic have the greatest impact on the low-temperature stability of asphalt mixture. The asphalt mixture is easy to fracture at a low temperature after ageing. The addition of fiber can inhibit the deterioration of low-temperature performance and induce crack resistance, reinforcement and toughening at low temperature. The correlation between ageing temperature and maximum bending strain is 0.8738, which is the second major factor. The increase in ageing temperature can deepen the ageing degree of asphalt mixture, resulting in poor low-temperature stability.

In terms of moisture stability, the influencing factors of ageing durability are ranked as follows: properties of fiber asphalt mastic > ageing temperature > aggregate gradation > ageing time. The correlation between the *TSR* value and properties of fiber asphalt mastic is 0.9790, indicating that properties of fiber asphalt mastic have the greatest impact on the moisture stability of asphalt mixture. Ageing aggravates the occurrence of moisture damage of asphalt pavement. The addition of fiber can not only delay the ageing of asphalt, but also improve the ability of asphalt mixture to resist freeze-thaw and moisture damage by its function of reinforcing and absorbing asphalt. Ageing temperature is the second major factor affecting the moisture stability and ageing durability of asphalt mixture. The increase in ageing temperature will accelerate the ageing of asphalt, decrease the content of oil components in asphalt, ultimately resulting in the decrease in cohesiveness and moisture stability.

(2) the influencing factors of freeze-thaw cycle durability of asphalt mixture are ranked as follows: properties of fiber asphalt mastic > freeze-thaw cycle times > aggregate gradation. The correlation between properties of fiber asphalt mastic and *TSR* value is 0.8279, which is the most major factor affecting the durability of freeze-thaw cycle. The freeze-thaw cycle failure of asphalt mixture is a process in which under the action of moisture and temperature, the bonding property of the interface between asphalt and aggregate is decreased, the structure of the material itself is destroyed, and the internal damage is gradually accumulated. The addition of fiber can effectively improve the viscosity and consistency of asphalt mastic, and the adsorption of fiber on asphalt enhances the freeze–thaw damage resistance of asphalt mixture.

(3) the influencing factors of fatigue durability of asphalt mixture are ranked as follows: aggregate gradation > properties of fiber asphalt mastic > stress ratio, but there is little difference between aggregate gradation and properties of fiber asphalt mastic. The correlation between aggregate gradation and fatigue life is 0.7744, proving that the intercalation and internal friction between aggregate particles and the adhesion between asphalt and aggregate have great influence on the fatigue durability of asphalt mixture. The correlation between properties of fiber asphalt mastic and fatigue life is 0.7687, indicating that the retarding effect of fiber on fatigue damage of asphalt mixture and the strengthening effect on the self-healing ability of asphalt mixture can effectively improve the fatigue durability of asphalt mixture.

In summary, the properties of fiber asphalt mastic have a great influence on the durability of asphalt mixture. The road plant fiber can effectively improve the properties of asphalt mastic and the durability of mixture. Combining the results in Section 3.2, Section 3.3 and Section 3.4, bamboo fiber has a better effect than lignin fiber in terms of improving and stabilizing the low-temperature ageing durability, moisture ageing durability, freeze-thaw cycle durability and resistance to repeated load of mixture.

## 4. Conclusions

(1) Under different ageing temperatures and times, the low-temperature ageing durability and moisture ageing durability of bamboo fiber asphalt mixture are better and more stable than those of lignin fiber asphalt mixture, but its mechanical properties are weaker than the latter. Fiber in asphalt mixture can make the stress of material more uniform and avoid stress concentration, improve compressive strength and decrease the resilient deformation. The rough surface and good oil absorption properties of bamboo fiber endow its modified asphalt mastic with good ductility and adhesion.
(2) Plant fiber can improve the integrity of asphalt mixture and weaken the effect of water erosion, thus improving the freeze-thaw cycle durability of asphalt mixture. The improvement effects of bamboo fiber and lignin fiber on freeze-thaw cycle durability of asphalt mixture are basically the same.
(3) Bamboo fiber has a higher density and better toughness, which is beneficial to improve the flexibility of the mixture and delay the development of cracks. Its modified asphalt mixture has good fatigue durability.
(4) Overall, the smaller the void ratio of fiber asphalt mixture, the thicker the asphalt film, the denser the structure, and the better its durability. The durability of the SMA gradation mixture is better than that of the AC gradation mixture.
(5) The material composition and aggregate gradation of fiber asphalt mixture have great impacts on its durability. In the future, it is necessary to establish a multiparameter comprehensive evaluation index system between fiber type and properties, asphalt mixture gradation and durability to achieve the directional regulation on the durability of different fiber asphalt mixtures.
(6) Bamboo fiber endows the asphalt mixture with good durability, presenting a viable alternative to lignin fiber. To facilitate future applications of asphalt pavements, the surface microstructure and chemistry of bamboo fiber and its dispersion in asphalt mixture can be further engineered or improved to unlock its potential.
(7) This study can lay the foundation for the practical application of bamboo fiber, and it is of great significance to improve the durability of asphalt pavement, reduce the project cost, promote the sustainable development of transportation in China, and protect the ecological environment.

## Figures and Tables

**Table 1:** Basic properties of SBS modified asphalt.

Property	Softening Point (°C)	Penetration (25 °C, 0.1 mm)	Ductility (5 °C, cm)	Relative Density	After TFOT * (163 °C, 5 h)		
					Mass Change (%)	Penetration Ratio (%)	Residual Ductility (5 °C, cm)
Measured value	81.1	48.2	37.1	1.030	−0.01	79.0	24
Specified value	≥pe	40~60	≥60	measured	≤1.0	≥65	≥15

* Note: thin film oven test.

**Table 2:** Basic properties of the two fibers used in this study.

Property	Length (mm)	Ash Content (%)	pH Value	Water Absorption (%)	Thermal Mass Loss Rate (210 °C, 2 h, %)	Oil Absorption (Times)	Relative Density (25 °C)
Lignin fiber	<5.5	13.2	7.2	4.7	5.7	7.3	0.897
Bamboo fiber	<6.0	15.0	7.1	4.4	5.5	9.2	0.943
Specified value	≤6.0	18 ± 5	7.5 ± 1.0	≤5	≤6.0	≥5 times the mass of the fiber	-

**Table 3:** Properties of aggregates used in this study.

Properties	Test Values of Aggregate with Each Size			Specified Value
	10–15 mm (Basalt)	5–10 mm (Basalt)	0–5 mm (Limestone)	
Apparent specific gravity	2.949	2.955	2.758	≥2.600
Bulk specific gravity	2.903	2.887	-	-
Water absorption (%)	0.55	0.80	-	≤2.60
Percent of flat and elongated particles (%)	4.7	11.3	-	≤15
Angularity (s)	-	-	48	≥30
Crushing value (%)	9.5	-	-	≤25
Los Angeles abrasion (%)	10.8	-	-	≤28

**Table 4:** Aggregate gradations of asphalt mixture (unit: %).

Mixture Type	Mass Fraction Pass Each Sieve (mm)										
	19	16	13.2	9.5	4.75	2.36	1.18	0.6	0.3	0.15	0.075
AC-13	-	100.0	95.5	80.0	46.5	35.8	26.0	19.3	14.0	10.6	7.4
AC-16	100.0	99.2	88.0	72.2	43.0	29.1	19.8	14.7	11.0	9.1	7.8
SMA-13	-	100.0	91.9	63.9	24.7	20.8	17.7	15.4	13.7	12.6	10.0
SMA-16	100.0	95.0	70.0	51.4	27.7	21.8	18.2	15.4	12.7	11.6	10.5

**Table 5:** Mix design data of each asphalt mixture.

MixtureType	Optimum Asphalt Content (%)	Optimum Fiber Content (%)	Gross Volume Density (g·cm^−3^)	Voidage (%)	Aggregate Voidage (%)	Asphalt Saturation (%)	Marshall Stability (kN)	Flow Value (mm)
AC-13	5.0	0.4	2.46	4.3	13.1	67.1	11.2	3.3
AC-16	4.9	0.4	2.45	4.5	13.9	67.9	11.3	3.1
SMA-13	5.9	0.4	2.51	3.9	16.8	74.5	8.8	2.1
SMA-16	5.7	0.4	2.45	4.3	16.9	75.0	8.6	2.3

**Table 6:** Optimum fiber and asphalt content of each fiber asphalt mixture.

Mixture Type	Optimum Fiber Content (%)	Optimum Asphalt Content (%)	
		Lignin Fiber	Bamboo Fiber
AC-13	0.4	5.0	5.3
AC-16	0.4	4.9	5.2
SMA-13	0.3	5.9	6.5
SMA-16	0.3	5.7	6.0

**Table 7:** Test results of physical properties of each fiber asphalt mastic.

Technical Index	Nonfiber Asphalt Mastic Asphalt Mortar	Lignin Fiber Asphalt Mastic	Bamboo Fiber Asphalt Mastic Asphalt Mortar
Softening point (°C)	81.1	87.3	86.6
Penetration (25 °C, 0.1 mm)	48.2	44.3	46.7
Ductility (5 °C, cm)	37.1	33.4	35.9
Density (g·cm^−3^)	1.030	1.021	1.024
Viscosity (135 °C, Pa·s)	7.89	8.86	9.05
Elastic recovery rate (%)	81	88	91

**Table 8:** Regression equation of fatigue life of asphalt mixture with different fibers.

Asphalt Type	Lignin Fiber	R^2^	Bamboo Fiber	R^2^	Nonfiber	R^2^
AC-13	y = −4.869x + 1.179 × 10^6^	0.9902	y = −5.157x + 1.597 × 10^6^	0.9921	y = −5.1572x + 6.210 × 10^5^	0.9935
AC-16	y = −5.122x + 6.596 × 10^5^	0.9921	y = −5.231x + 7.534 × 10^5^	0.9951	y = −5.3763x + 4.854 × 10^5^	0.9930
SMA-13	y = −3.983x + 1.570 × 10^6^	0.9909	y = −4.096x + 1.854 × 10^6^	0.9950	-	-
SMA-16	y = −4.491x + 1.343 × 10^6^	0.9998	y = −4.601x + 1.782 × 10^6^	0.9997	-	-

**Table 9:** Evaluation indexes and influence parameters of ageing durability of fiber asphalt mixture.

Performance Index	Serial Number	Compressive Strength (MPa)	Maximum Bending Strain (×10^−6^ με)	*TSR* (%)	Ageing Temperature (°C)	Ageing Time (h)	4.75 mm Pass Rate (%)	Penetration (25 °C, 0.1 mm)	Ductility (5 °C, cm)	Viscosity (135 °C, Pa·s)
Mechanical properties	1	5.56	-	-	100	30	46.5	48.2	-	-
	2	5.82	-	-	90	0	37	44.3	-	-
	3	7.55	-	-	80	60	24.7	46.7	-	-
	4	5.84	-	-	120	120	27.7	44.3	-	-
	5	7.15	-	-	100	30	24.7	46.7	-	-
Low- temperature stability	1	-	2688.1	-	100	30	46.5	-	37.1	-
	2	-	3155.4	-	90	0	37	-	33.4	-
	3	-	3226.4	-	80	60	24.7	-	35.9	-
	4	-	2307.8	-	120	120	27.7	-	33.4	-
	5	-	3160.8	-	100	30	24.7	-	35.9	-
Moisture stability	1	-	-	80.16	100	30	46.5	-	-	7.89
	2	-	-	91.89	90	0	37	-	-	8.86
	3	-	-	93.68	80	60	24.7	-	-	9.05
	4	-	-	80.84	120	120	27.7	-	-	8.86
	5	-	-	91.45	100	30	24.7	-	-	9.05

**Table 10:** Evaluation indexes and influence parameters of freeze-thaw cycle durability and fatigue durability of fiber asphalt mixture.

Performance Index	Serial Number	*TSR* (%)	Fatigue Life (Times)	Freeze-Thaw Times (Times)	Viscosity (135 °C, Pa·s)	4.75 mm Pass Rate (%)	Stress Ratio	Elastic Recovery Rate (%)
Freeze-thaw cycle durability	1	66.33	-	5	7.89	46.5	-	-
	2	79.88	-	3	8.86	37.0	-	-
	3	93.05	-	2	9.05	24.7	-	-
	4	95.93	-	1	8.86	27.7	-	-
	5	81.31	-	5	9.05	24.7	-	-
Fatigue durability	1	-	4024	-		46.5	0.4	81
	2	-	1616	-		37.0	0.5	88
	3	-	9291	-		24.7	0.4	91
	4	-	3220	-		27.7	0.5	88
	5	-	32,907	-		24.7	0.3	91

## Data Availability

Not applicable.
